# Dystonia updates: definition, nomenclature, clinical classification, and etiology

**DOI:** 10.1007/s00702-021-02314-2

**Published:** 2021-02-19

**Authors:** Karen Grütz, Christine Klein

**Affiliations:** Institute of Neurogenetics, University of Lübeck, Ratzeburger Allee 160, 23538 Lübeck, Germany

**Keywords:** Dystonia, Clinical classification, Disease etiology, Nomenclature

## Abstract

A plethora of heterogeneous movement disorders is grouped under the umbrella term dystonia. The clinical presentation ranges from isolated dystonia to multi-systemic disorders where dystonia is only a co-occurring sign. In the past, definitions, nomenclature, and classifications have been repeatedly refined, adapted, and extended to reflect novel findings and increasing knowledge about the clinical, etiologic, and scientific background of dystonia. Currently, dystonia is suggested to be classified according to two axes. The first axis offers precise categories for the clinical presentation grouped into age at onset, body distribution, temporal pattern and associated features. The second, etiologic, axis discriminates pathological findings, as well as inheritance patterns, mode of acquisition, or unknown causality. Furthermore, the recent recommendations regarding terminology and nomenclature of inherited forms of dystonia and related syndromes are illustrated in this article. Harmonized, specific, and internationally widely used classifications provide the basis for future systematic dystonia research, as well as for more personalized patient counseling and treatment approaches.

## Introduction

Dystonia is the third most common movement disorder after Parkinson’s disease and essential tremor. International efforts in patient recruitment, rating scale use and harmonization, increasing scientific background on etiology and pathophysiology, novel therapeutic approaches, and, last but not least, the engagement of patients themselves, have (re-)shaped our understanding and awareness of dystonia and related syndromes in recent years. In light of its broad clinical and etiological heterogeneity, it becomes obvious that suitable definitions, a widely accepted and commonly used nomenclature, as well as uniform classifications of dystonic syndromes are a key prerequisite to (i) effectively communicate in the scientific community and with patients and caregivers; (ii) aide in establishing a clinically and/or etiologically defined diagnosis, and (iii) provide an important framework for the study of known and newly identified forms of dystonia or syndromes with dystonia as a prominent feature. The constant extension of knowledge and data prompts the need for necessary adaptations to the current nomenclature and classification schemes. In the following sections, we will present the most recent definitions, nomenclature, and consensus updates regarding clinical classification and etiology.

### Definition of dystonia

Patients suffering from “dystonia musculorum deformans” were published in 1911 by Oppenheim (Oppenheim 1911; Klein and Fahn 2013), who thus coined the term dystonia. The aforementioned individuals presented with muscle spasms leading to twisted postures that were more severe upon walking. These spasms or movements were described as rapid and rhythmic. Furthermore, the symptoms were of progressive nature and the muscle tone fluctuated from hypotonic to tonic (Oppenheim 1911; Klein and Fahn 2013). Oppenheim even pointed out that all individuals shared the same geographic and ethnic background (Ashkenazi Jews) indicating an inherited disorder. Over the following years, a multitude of patients were described with different forms of what would today be called dystonia.

Still, it took more than 60 years from Oppenheim’s first description of dystonia for the First International Dystonia Symposium to take place in 1975. This opportunity was used to reassess the clinical presentations of focal dystonia, such as blepharospasm, spasmodic dysphonia, torticollis, oromandibular dystonia, and writer’s cramp. In the following years, it was suggested to define dystonia as the umbrella term for these heterogeneous disorders (Marsden 1976a, b; Sheehy and Marsden 1982). Still, a crucial issue was the development of a coherent and systematic definition of dystonia and associated syndromes, especially upon the identification of (novel) genetic contributions and complex phenotypes. The first consensus definition of dystonia was established by an assigned committee of the Dystonia Medical Research Foundation in 1984. Here, the syndrome was defined to consist “of sustained muscle contractions, frequently causing twisting and repetitive movements, or abnormal postures” (Fahn et al. 1987). However, the quickly growing body of information on clinical presentations and etiological factors of dystonia required frequent adjustment of definitions. Over the past ~ 35 years, several limitations of previous definitions have been identified and, based on a consensus statement of a Task Force of the International Parkinson and Movement Disorder Society, the revised definition states in 2013: “Dystonia is a movement disorder characterized by sustained or intermittent muscle contractions causing abnormal, often repetitive, movements, postures, or both. Dystonic movements are typically patterned, twisting, and may be tremulous. Dystonia is often initiated or worsened by voluntary action and associated with overflow muscle activation” (Albanese et al. 2013). Identification of an increasing number of dystonia genes led to further refinement of the classification to facilitate establishing a specific diagnosis and genetic testing and counseling of dystonia patients. This most recent and currently used classification scheme reduces the number of conceptional axes to two, combining age at onset and body distribution as clinical characteristics, which also includes the temporal pattern and further associated features. The second axis concerns the etiology with respect to pathology of the nervous system as well as inheritance and other acquisitions of the disease (Albanese et al. 2013).

### Nomenclature

Whenever a group of disorders, in this case dystonia, and associated features reach a certain level of complexity in the way they are presenting, it is inevitable to refer to these phenotypes with a precise and uniformly used terminology. This is not only beneficial for the involved neurologists and other physicians caring for patients with dystonia, but also for the patients themselves. There are numerous ways to categorize different subgroups of a disorder and these classifications have the tendency and also necessity to evolve over time. Classifications can be organized according to clinical presentation, etiology or any other nominator that is of relevance for the respective disorder. The terms isolated, combined and complex dystonia, for example, provide immediate insight into the clinical picture and whether the dystonic presentation is the sole phenotype or connected with other features, possibly even as part of a different underlying condition.

For types of dystonia with a suspected genetic etiology, originally, the designation “DYT”, i.e. DYT1, was introduced to catalogue chromosomal regions that had been linked to a familial disorder, while the actual underlying gene was still unidentified (Kramer et al. 1990). Multiple problems regarding this system of designation have accumulated over time, including, but not limited to, multiple designations for the same disorder or no further confirmation of an identified locus or later gene (Marras et al. 2012). To establish a reference list for all genetically determined movement disorders, the International Parkinson and Movement Disorder Society (MDS) Task Force for Nomenclature of Genetic Movement Disorders recommends the following criteria for a designation assignment (Marras et al. 2016): (i) Disorders should only receive a designation when genetic testing of the known gene or haplotype is possible. (ii) The phenotype prefix has to be appropriately chosen and the phenotype has to be confirmed by two independent groups. The prefix should refer to the most prominent phenotype of the disorder, i.e. DYT in the case of dystonia. In cases with two equally prominent features, a double prefix can be chosen as is the case for DYT/PARK-*ATP1A3*. (iii) With respect to the errors in the numerical listing and the identification of further increasing numbers of causative genes, the number suffixes should be replaced with the gene name. As an example, for early-onset generalized dystonia caused by mutations in the *TOR1A* gene, the designation has changed from DYT1 to DYT-*TOR1A*. (iv) A designation should only be assigned for monogenic disorders, while risk factor genes are to be listed separately. (v) A certain threshold of evidence for a genotype–phenotype correlation has to be reached to assign a locus symbol. Besides a designation system for the genetically confirmed cases, a uniform classification is of high relevance for all forms of dystonia and, thus, the relevant nomenclature will be explained in the following sections in the respective classification context.

### Clinical classification

**Fig. 1:**
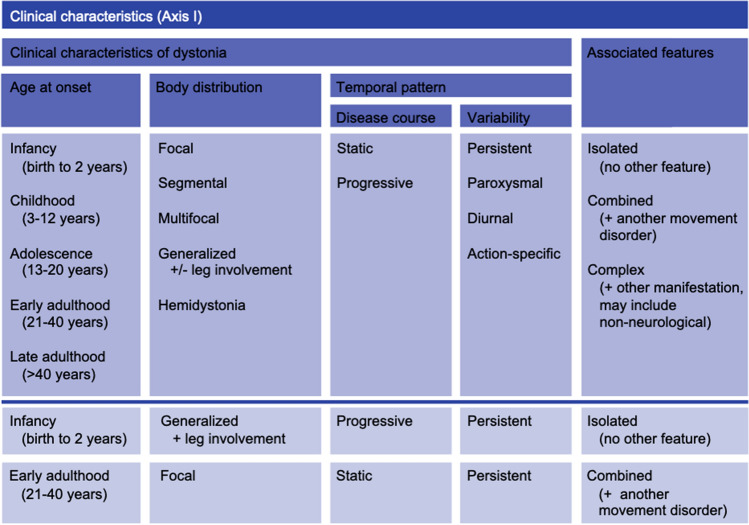
Clinical characteristics (Axis I) (adapted from Albanese et al. 2013). In the upper part of the scheme, the four descriptors age at onset, body distribution, temporal pattern, and associated features are depicted with additional categories (temporal pattern) and all recommended subcategories. In the lower part, below the dark blue line, two sets of clinical descriptors are given as examples that are frequently seen in the clinic, although all other combinations of descriptions are possible, as well

A set of four descriptors can and should be used to illustrate the phenomenology of dystonia, i.e. age at onset, body distribution, temporal pattern, and associated features. The clinical classification or Axis I, as described by Albanese et al. (2013), is schematically shown in Fig. 1. The clinical descriptors will be detailed in the following paragraphs.

### Age at onset

**Fig. 2:**
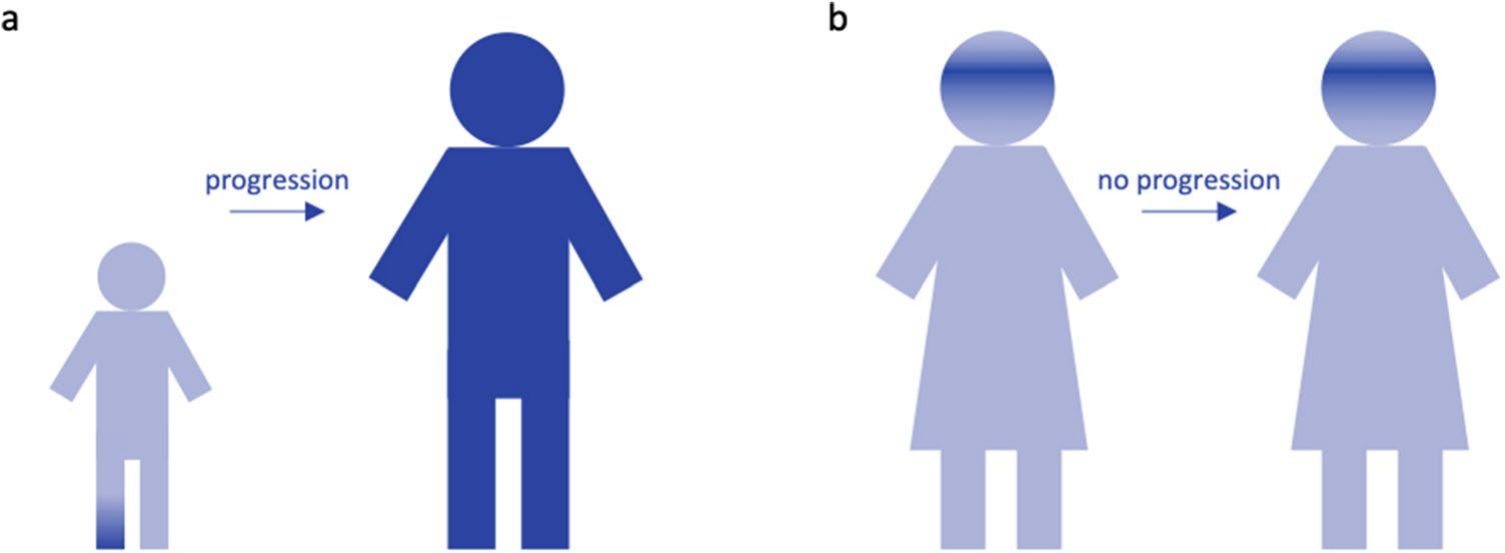
Disease progression. Dark blue indicates body regions affected with dystonia. Children presenting with focal dystonia have a higher tendency to progress to generalized dystonia (**a**), while individuals with a late onset, in this case a woman with blepharospasm, will commonly remain stable in their dystonic presentation (**b**)

Broadly accepted, the age at onset is of great importance for the establishment of the diagnosis of a specific form of dystonia, as well as for patient counselling and care, due to its prognostic value. Children suffering from dystonia are more likely to have a detectable cause and a tendency of the dystonia to generalize, whereas dystonic signs in adulthood are more likely to remain focal (Fig. 2). These two examples display the two most extreme examples in the broad spectrum of disease presentation in combination with phenotypic progression.

The observation that different phenotypes present within distinct age groups has led to the use of age at onset as a nominal classification. In this sense, three age groups had been suggested in earlier classifications: childhood (0–12 years), adolescent (12–20 years), and adult onset (> 20 years) (Fahn 1988). In the following adaptations, even more refined categories seemed appropriate as, for example, the appearance of dystonia before the age of 1 year is most likely due to an inherited disorder (Sanger 2003). Therefore, Albanese and colleagues have suggested a scheme to cluster age at onset in a similar fashion as it is being done for other neurological disorders: (i) infancy (birth to 2 years), (ii) childhood (3–12 years), (iii) adolescence (13–20 years), (iv) early adulthood (21–40 years), (v) late adulthood (> 40 years) (Albanese et al. 2013). For some types of dystonia, the age at onset may be covering two (or more) of these clusters, as the phenotypic variability cannot always be forced into such rather fixed borders. When focusing on monogenic forms of dystonia, age clusters are observed for individual forms of dystonia. DYT-*TOR1A*, for instance, has a median age at onset of 9 years (childhood), whereas age at onset in DYT-*GNAL* would be classified at the upper end of the early adulthood group with a median age at onset of 38 years (MDS Gene, www.mdsgene.org). When grouping the forms of monogenic isolated dystonia together, the median age at onset ranges from 6 to 38 years, while the combined forms range from 0 to 40 years in terms of age at onset (MDS Gene, www.mdsgene.org). This additionally highlights how important it is to relate the clinical description to age at onset of a patient to identify patterns or certain characteristics that will help establish a specific diagnosis or treatment.

### Body distribution

Dystonic signs can present in any given region of the body. Usually, the cranial and cervical region, the larynx and trunk as well as the limbs are affected, either individually or in any given combination. Diagnosis and also effective therapy vastly depend on the classification of symptomatic body regions. Despite the complexity and magnitude of dystonia subtypes, effective treatments, including oral medications, botulinum toxin, and surgical interventions are available for the majority of patients (Jinnah 2020).

Furthermore, the clinical description of involved regions might help predict motor symptoms in the course of the disease. The pattern of distribution my change over time, leading to involvement of additional dystonic regions. The following classifications should be considered according to the latest consensus update (Albanese et al. 2013):

*“Focal Dystonia*: Only one body region is affected. Typical examples of focal forms are blepharospasm, oromandibular dystonia, cervical dystonia, laryngeal dystonia, and writer’s cramp. Cervical dystonia is considered a form of focal dystonia, although by convention, the shoulder can be included as well as the neck.

*Segmental Dystonia*: Two or more contiguous body regions are affected. Typical examples of segmental forms are: cranial dystonia (blepharospasm with lower facial and jaw or tongue involvement) or bibrachial dystonia.

*Multifocal Dystonia*: Two noncontiguous or more (contiguous or not) body regions are involved.

*Generalized Dystonia*: The trunk and at least two other sites are involved. Generalized forms with leg involvement are distinguished from those without leg involvement.

*Hemidystonia*: More body regions restricted to one body side are involved. Typical examples of hemidystonia are due to acquired brain lesions in the contralateral hemisphere.”

### Temporal pattern

Signs and severity can vary widely over time. For instance, dystonia can spread, as mentioned in the previous section, which allows for a distinction of static and progressive forms. The phenomenology can show momentary and diurnal variability and fluctuate from day to day or during the course of a single day, as seen in dopa-responsive dystonia. The variability of the phenotype also allows distinguishing forms with a consistent occurrence, i.e. task or action-specific dystonia or dystonia at rest, from trigger-induced variable forms (e.g. paroxysmal dystonia). Further factors modifying the phenotype in addition to voluntary actions and external triggers are compensatory phenomena, gestes antagonistes (or alleviating movements) or psychological state (Albanese et al. 2013). Clinically, the temporal pattern is especially important to define a specific diagnosis and also treatment options. Temporal phenotypic variability can be categorized into four groups:

*Persistent*: This describes dystonia present throughout the day with roughly the same intensity.

*Paroxysmal*: Episodes of dystonia are self-limited and typically induced by a trigger. After the episode, the patient returns into the previous neurological state.

*Diurnal fluctuations*: Phenomenology, severity and presence of dystonia vary following obvious circadian rhythm.

*Action-specific*: Dystonic movements that are only present while execution a very specific task.

### Associated features

Dystonia can be the sole phenotype, or it might occur in conjunction with other movement disorders. As such, forms of dystonia combined with myoclonus, parkinsonism, or other movement disorders have been termed as defined syndromes. Previously, syndromes manifesting only with dystonia have been considered “primary” (Fahn et al. 1998; Fahn 2011). The term “primary” is widely used in other disorders, meaning either that the condition presented as first sign, that it is the major subgroup, or to describe that no other irregularity has been detected (Webster’s New World Medical Dictionary 2008). It is thus recommended to use terms that unambiguously describe the clinical presentation without having a connotation regarding the etiology of the disease (Albanese et al. 2013). For this reason and further extending beyond a dichotomous scheme of isolated vs. combined dystonia, we would like to suggest the use of three terms, i.e. *isolated*, *combined*, and *complex dystonia*, each of which will be featured in accompanying reviews of this issue (Domingo et al. 2020; Weissbach et al. 2020; Herzog et al. 2020). Isolated dystonia describes phenotypes, where dystonia is the sole motor feature, with the exception of tremor. In the case of combined dystonia, other movement disorders, i.e. parkinsonism, myoclonus, or dyskinesia, present in conjunction with dystonia (Klein et al. 2017). Of note, the combination of dystonia with non-motor features has also been reported in multiple instances (Novaretti et al. 2019; Timmers et al. 2019; Ferrazzano et al. 2019). *Complex dystonia* describes syndromes composed of dystonia in conjunction with other neurologic or systemic presentations. In many of these syndromes, dystonia may only be an inconsistent feature or not the most prominent disease manifestation and there is wide phenotypic variability with respect to dystonia across individual patients. These syndromes include, but are not limited to, a range of neurodegenerative diseases, disorders leading to brain calcification, disorders of heavy metal metabolism, neurodegeneration with brain iron accumulation (NBIA), lipid storage disorders, mitochondrial disorders, organic acidurias, and disorders of thiamine metabolism (Klein et al. 2017). In Wilson disease, for example, dystonia presents in conjunction with neurological or psychiatric features, as well as a dysfunctional liver (Rosencrantz and Schilsky 2011).

**Fig.3:**
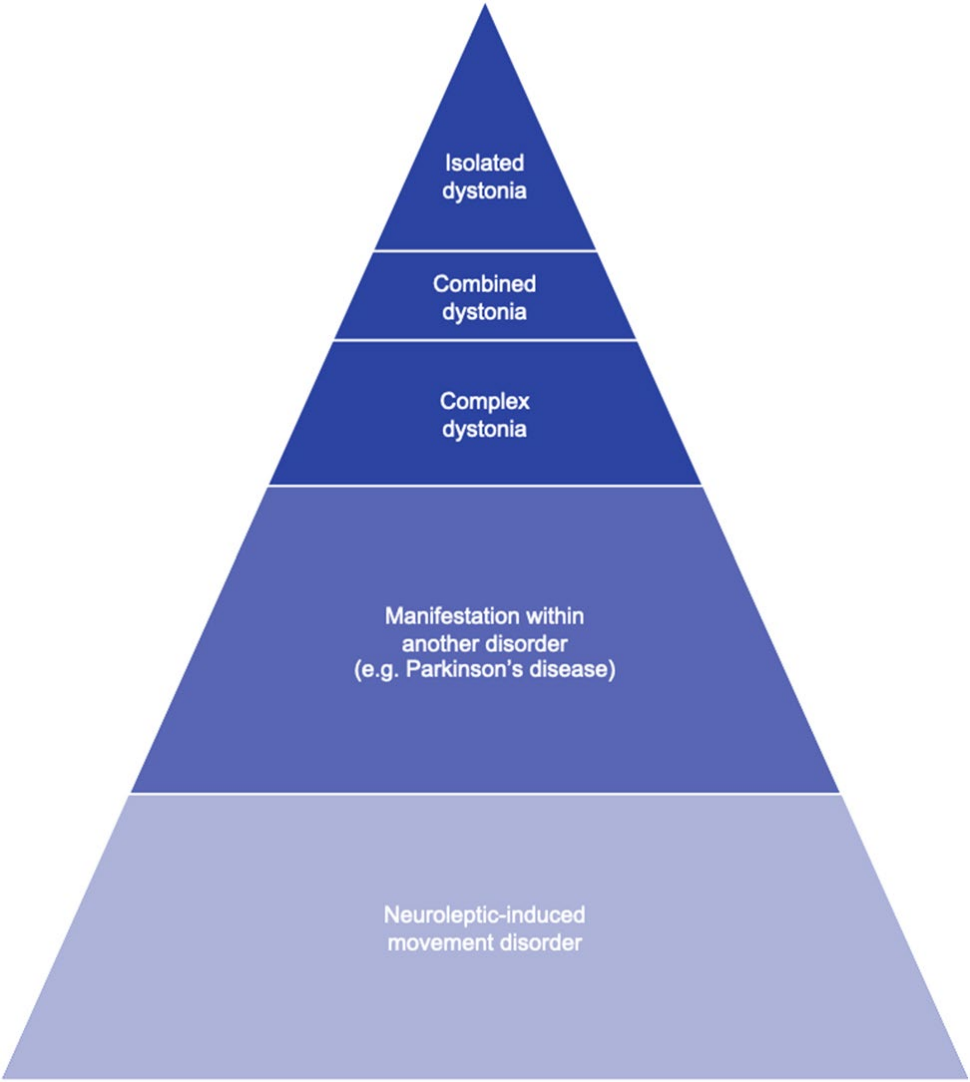
Schematic overview on the occurrence of dystonia as a clinical sign according to frequency regardless of the underlying primary condition

Although the focus of the present article is on dystonia and dystonic syndromes per se (i.e. isolated combined, and complex forms of dystonia), it should be noted that, when it comes to the mere frequency of dystonic signs irrespective of the primary underlying condition, dystonia most commonly occurs as a clinical sign within other, more common conditions, such as Parkinson’s disease, or as a side effect of drugs used to treat psychiatric disorders (Mulroy et al. 2020). This consideration is schematically illustrated in Fig. 3.

### Etiology

**Fig. 4:**
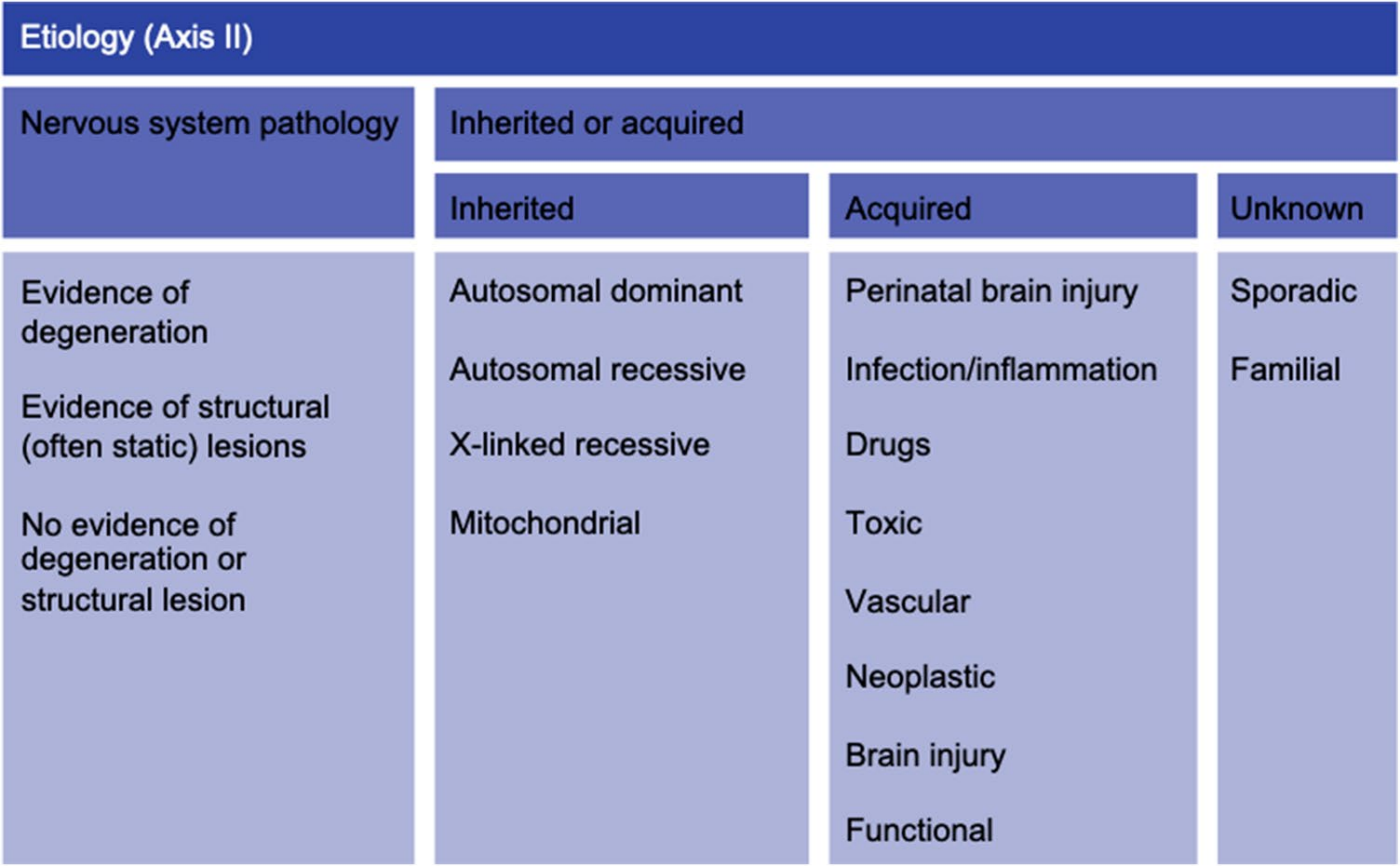
Etiology (Axis II) (adapted from Albanese et al. 2013). The etiological axis is subdivided into the contribution of the nervous system and the presence of genetic or acquired origin leading to development of dystonia. As an example, an etiological description of a dystonia case can be ‘evidence of degeneration’ and an ‘X-linked recessive inheritance pattern’, as it the case for X-linked dystonia parkinsonism (DYT/PARK-*TAF1*)

The second axis of classification is based on the etiological background of dystonia. A schematic representation is depicted in Fig. 4. Even though our understanding of the etiology of dystonia has evolved over time, for most forms it still cannot be fully explained. With new information, clinical, genetic and from basic science, the classification of dystonia based on etiology will have to be constantly adapted. The term “primary” which was or still is used as an etiological category for genetically confirmed isolated dystonia without other pathological findings (Fahn et al. 1998), is no longer suggested (Albanese et al. 2013).

### Pathological findings of the nervous system in dystonia

In keeping with its heterogeneity and broad spectrum including both degenerative and (likely) non-degenerative conditions, there is no uniform anatomical description of dystonia. Several studies suggest that there is no apparent macroscopic degeneration or irregularity in the brain in isolated dystonia (Rostasy et al. 2003; Paudel et al. 2012, 2016). This is different in forms of dystonia with a neurodegenerative pattern, such as DYT/PARK-*TAF1* (Hanssen et al. 2019). Neuroimaging studies, on the other hand, have identified more subtle alterations, with respect to cortical thickness and gray matter volume differences in cortical regions, basal ganglia, thalamus, hippocampus, and amygdala in focal dystonia (Tomić et al. 2020), as well as for the subthalamic area of the brain stem in myoclonus-dystonia (van der Meer et al. 2012). Other studies have identified cellular alterations in specific forms of dystonia, such as neuronal inclusions in brainstem nuclei in patients with DYT-*TOR1A* (McNaught et al. 2004), suggesting abnormalities on the cellular level. These findings still need further replication and extension, as is true for another line of research linking dystonia to cerebellar dysfunction based on Purkinje cell loss and axonal swelling in the cerebellum of patients suffering from cervical dystonia (Prudente et al. 2013). Recently, it has been shown that the genetic reduction of *Lpin1* in a *Tor1a* mouse model had a positive effect on survival but also suppressed motor dysfunction and nuclear membrane pathology (Cascalho et al. 2020). The molecular pathophysiological construct in dystonia will be more detailed in an accompanying review (Gonzalez-Latapi et al. 2021).

With more studies possibly identifying cellular alterations with more specific deficits, the terminology of pathological changes will have to be reconsidered and adapted in future classifications of dystonia. Nevertheless, the identification of degeneration, be it macroscopic, microscopic or on a molecular basis, provides a valuable discriminator for different forms of dystonia with respect to pathological abnormalities. According to the latest consensus update, the first subgroup is considered to show degeneration, with a progressive abnormality, e.g. neuronal loss. The second group shows static lesions, either non-progressive anomalies or acquired lesions, and the third subgroup displays no evidence of degeneration or structural lesions (Albanese et al. 2013).

### Inherited dystonia

Inherited forms of dystonia require a confirmed genetic origin and can again be subdivided into multiple groups according to the pattern of inheritance.

*Autosomal dominant*: Several forms, such as DYT-*TOR1A* (Ozelius et al. 1997), DYT/PARK-*GCH1* (Ichinose et al. 1994; Segawa et al. 2003), DYT-*THAP1* (Fuchs et al. 2009), DYT-*SGCE* (Zimprich et al. 2001), and DYT/PARK-*ATP1A3* (De Carvalho Aguiar et al. 2004), fall into the category of autosomal dominantly inherited dystonia.

*Autosomal recessive*: This subgroup includes forms, such as DYT-*ATP7B*, also known as Wilson disease (Bull et al. 1993), NBIA/DYT-*PANK2* or pantothenate kinase-associated neurodegeneration (PKAN) (Zhou et al. 2001), and NBIA/DYT/PARKa-*PLA2G6* or *PLA2G6-*associated neurodegeneration (PLAN) (Morgan et al. 2006). Also, multiple metabolic disorders can be found in this category.

*X-linked recessive*: This subgroup contains disorders, such as DYT/PARK-*TAF1* (Makino et al. 2007), DYT/CHOR-*HPRT* or Lesch-Nyhan syndrome (Gibbs and Caskey 1987), and DYT-*TIMM8A*, also known as Mohr-Tranebjaerg syndrome (Tranebjærg et al. 2000) which are all inherited in an X-chromosomal fashion.

*Mitochondrial*: Inherited forms with mutations in the mitochondrial genome are, for example, Leigh syndrome or DYT-mt-*ND6* (Leber optic atrophy and dystonia) (Kim et al. 2010).

Notably, a large proportion of the recessive forms (autosomal and X-linked) as well as the mitochondrial forms are classified as complex dystonia forms, whereas all isolated dystonias with a known genetic causality are inherited in an autosomal dominant fashion (Klein et al. 2017).

### Acquired dystonia

Several causal factors for the acquisition of dystonia have been documented so far. A useful categorization is illustrated in the following list adapted from (Albanese et al. 2013):

*Perinatal brain injury*: dystonic cerebral palsy, delayed-onset dystonia.

*Infection/inflammation*: viral encephalitis, encephalitis lethargica, subacute sclerosing panencephalitis, human immunodeficiency virus (HIV) infection, autoimmune causes, other (tuberculosis, syphilis, etc.)

*Drugs*: levodopa and dopamine agonists, neuroleptics (dopamine receptor blocking drugs), anticonvulsants, and calcium channel blockers.

*Toxic*: manganese, cobalt, carbon disulfide, cyanide, methanol, disulfiram, and 3-nitropropionic acid.

*Vascular*: ischemia, hemorrhage, and arteriovenous malformation (including aneurysm).

*Neoplastic*: brain tumor, and paraneoplastic encephalitis.

*Brain injury*: head trauma, brain surgery (including stereotactic ablations), and electrical injury.

*Functional*

### Dystonia of unknown etiology

Dystonia with an unknown cause, can be further divided into *sporadic* and *familial* forms. In case of familial forms, it seems likely that there is a genetic contribution. With the discovery of novel dystonia genes, such as *GNAL*, *ANO3*, *KCTD17*, and *KMT2B* (Charlesworth et al. 2012; Fuchs et al. 2013; Mencacci et al. 2015; Meyer et al. 2017), these subtypes can now be allocated to the realm of inherited forms of dystonia.

### Additional observations and perspectives

**Fig. 5:**
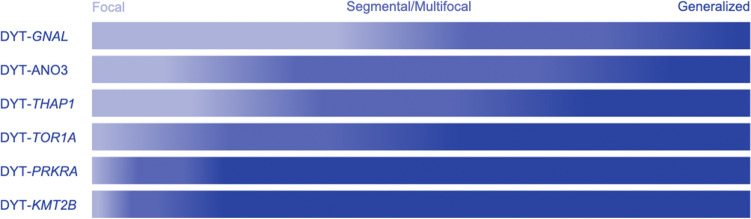
Body distribution with respect to genetic background. Different shades of blue indicate the different clinical presentations with darker tones relating to more severe presentations. The fractions are in accordance with the numbers reported by Lange et al. (2021)

The current classifications combine the most important observations that have been made concerning dystonia. Nevertheless, not all pieces of information have been included or even identified. As previously shown, an important point is the constant adaptation of any classification scheme according to the most current knowledge. This also includes an engaged exchange of information between clinicians, scientists, caregivers, and last but not least, patients, as they benefit from as specific a diagnosis and refined a prognosis of their disorder as possible. As an example, recent findings have identified patterns of symptom spreading in adult-onset isolated focal dystonia (Berman et al. 2020). However, even with knowledge about the genetic origin of a disease, an individual prognosis can be difficult, if not impossible due to the broad phenotypic spectrum, sometimes even within families. Still, at a group level, certain patterns with regard to body distribution of dystonic features can be recognized when comparing different monogenic forms of dystonia (Fig. 5).

Of translational relevance, testing guidelines can be refined upon identification of clinical patterns (Bressman et al. 2000). First, a clinical categorization can lead to better defined genetic testing, second, a specific diagnosis can help determine a progression pattern and, third, may also allow for a better prediction of response to treatment, such as deep brain stimulation, dopaminergic medication, etc. (Jinnah et al. 2018). Thus, treatment response may offer yet another aspect of classification.

In addition to already known genetic causes of dystonia, genetic risk factors could also play a role in disease etiology and may become important for classificational purposes in the future. Due to the heterogeneity of dystonia and associated syndromes, as well as its overall rarity, no large genetic association studies have been conducted to date. In more defined groups of patients, i.e. isolated dystonia, several studies, almost exclusively candidate-based, have been performed but no compelling association has been identified (Ohlei et al. 2018). Furthermore, variants in several molecular pathways have been detected, however, with the necessity to confirm a robust association in larger cohorts (Siokas et al. 2018).

Assembly of data on dystonia patients (on a national or even international) level has evolved greatly over the years. This has led to the ability to conduct large cross-sectional studies that will enable the identification of factors influencing the prevalence and phenomenology of clinical characteristics, such as tremor, in dystonia (Shaikh et al. 2020).

**Fig. 6:**
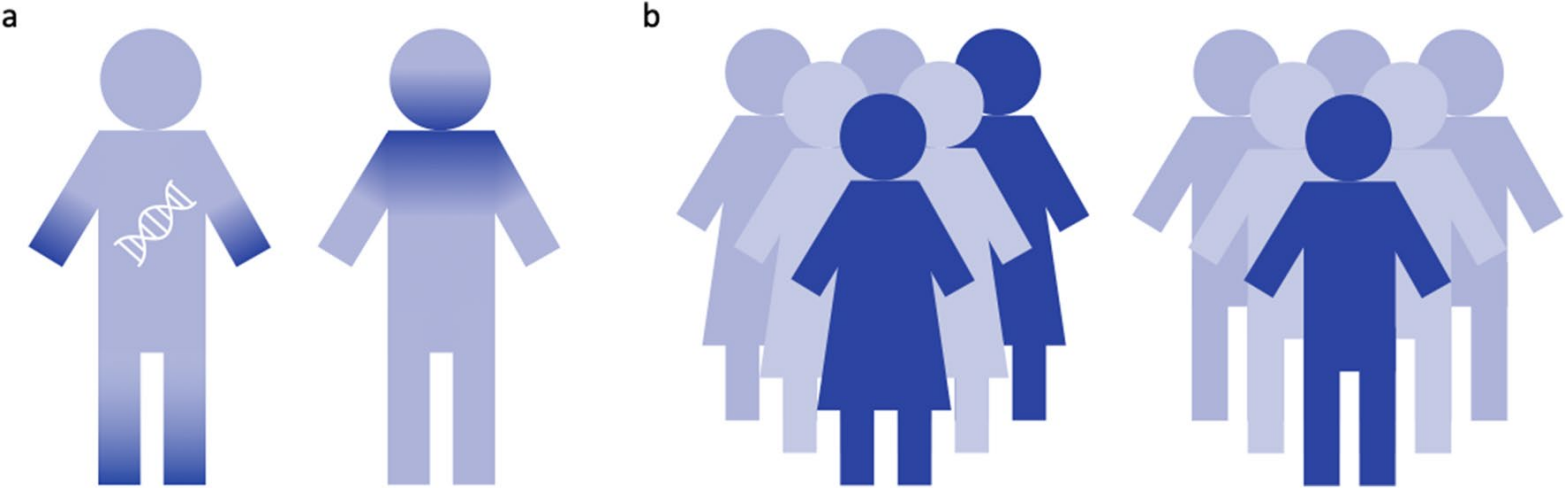
Distribution of signs and gender differences. **a** Affected body regions are shown in dark blue. Dystonia of the extremities is more likely to have a genetic origin. **b** Dark blue represents affected individuals. Women are overall more likely to develop dystonic signs than men

Genetic forms, while being of great importance to disease modeling using basic science approaches, only explain the disease in a minor proportion of cases. The majority of patients will not have a monogenic origin of dystonia. Importantly, however, there are patterns linking the likelihood of a genetic origin to certain phenotypic expressions. For example, it is more likely to identify a genetic cause in patients with dystonia of the extremities versus those suffering from a cranial form (Fig. 6a). Equally compelling is the observation that, overall, more women are affected with dystonia in comparison to men (Epidemiologic Study of Dystonia in Europe (ESDE) Collaborative Group 1999) (Fig. 6b). When analyzing individual subgroups of dystonia, however, also the reverse scenario is possible with an excess of affected males in most focal task-specific dystonias (Meoni et al. 2020), highlighting the as yet poorly understood influence of gender on the development of dystonia.

Even more complex systems biology approaches may lead to the development of further means of categorization of dystonia patients. A recent study has modeled the contribution of dystonia-associated genes to dystonia pathology (Mencacci et al. 2020). This study also highlights that both types of studies, candidate-based genetic and functional studies as well as hypothesis-free studies, will likely contribute to a more refined subgrouping of the dystonias in the future.

**Fig. 7:**
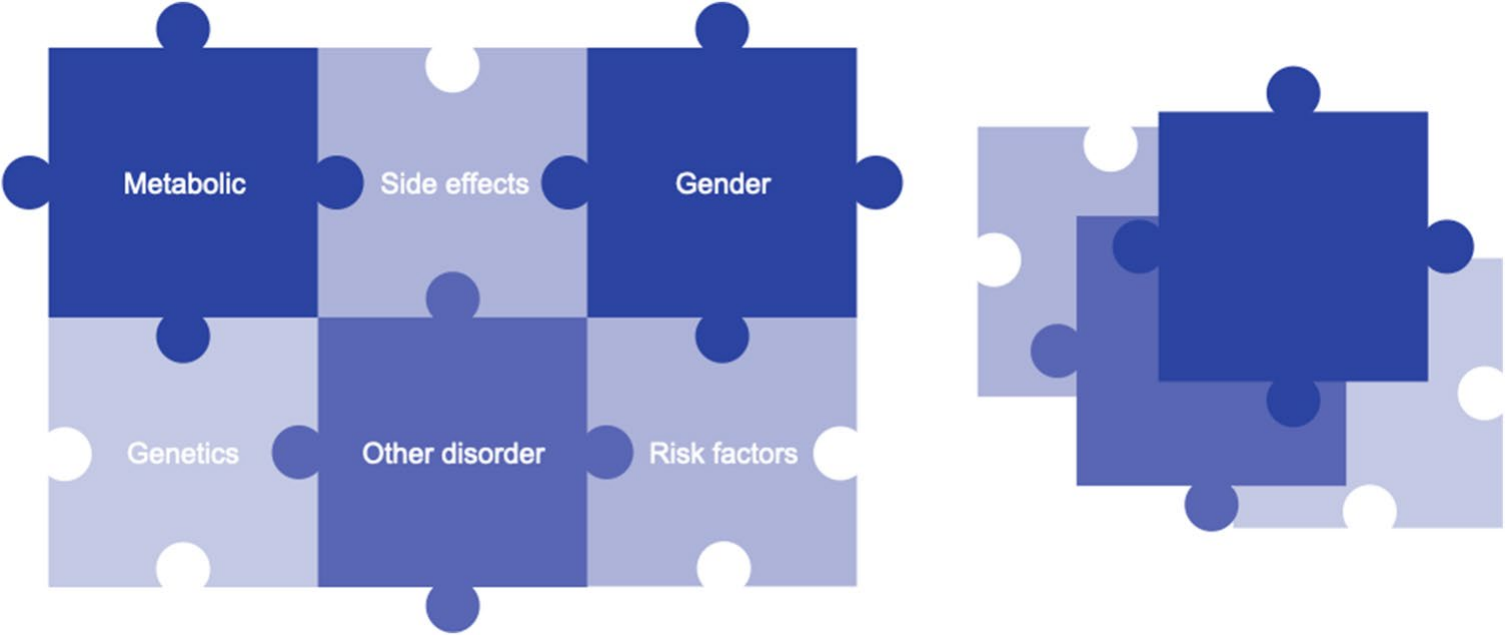
Factors contributing to the development and expression of dystonia. Several parts of the puzzle have already been assembled, whereas other pieces of yet unknown entities still need to be added

In conclusion, all of the above-mentioned definitions, terms, and classification schemes are of tremendous importance for three reasons: (i) They can be used to comprehensively and specifically describe the spectrum of signs and related information of any (dystonia) patient to all involved parties, i.e. the patient, clinicians, caregivers, but also geneticists and scientists. (ii) The classifications enable the detection of certain patterns and thus facilitate the establishment of a specific diagnosis. (iii) Existing classifications can be the starting point for future refinements and additions, stemming from scientific discoveries in clinical research, basic science and system biology approaches, genetic analyses, and treatment development. Ultimately, the identification and integration of all of these pieces of the puzzle (Fig. 7) will lead to an improved understanding of dystonia and patient care.
